# Longitudinal Rise in Seroprevalence of SARS-CoV-2 Infections in Children in Western Germany—A Blind Spot in Epidemiology?

**DOI:** 10.3390/idr13040088

**Published:** 2021-11-12

**Authors:** Folke Brinkmann, Hans H. Diebner, Chantal Matenar, Anne Schlegtendal, Jan Spiecker, Lynn Eitner, Nina Timmesfeld, Christoph Maier, Thomas Lücke

**Affiliations:** 1 University Children’s Hospital, Ruhr-University Bochum, 44791 Bochum, Germany; folke.brinkmann@rub.de (F.B.); chantalmatenar15@gmail.com (C.M.); anne.schlegtendal@rub.de (A.S.); jan.spiecker@gmail.com (J.S.); lynn.eitner@rub.de (L.E.); christoph.maier@rub.de (C.M.); 2 Department of Medical Informatics, Biometry and Epidemiology, Ruhr-University Bochum, 44801 Bochum, Germany; hans.diebner@rub.de (H.H.D.); nina.timmesfeld@amib.rub.de (N.T.)

**Keywords:** SARS-CoV-2, children, seroprevalence

## Abstract

SARS-CoV-2 infection rates in children and adolescents are often underestimated due to asymptomatic or oligosymptomatic infections. Seroprevalence studies can reveal the magnitude of “silent” infections in this age group and help to assess the risk of infection for children but also their role in spreading the disease. In total, 2045 children and their parents from the Ruhr region were finally included after the exclusion of drop-outs. Seroconversion rates among children of all age groups increased from 0.5% to 8% during the study period and were about three to fourfold higher than the officially registered PCR-based infection rates. Only 41% recalled symptoms of infection; 59% were asymptomatic. In 51% of the infected children, at least one parent also developed SARS-CoV-2 antibodies. Depending on local incidences, the rates of seroconversion rose to different levels during the study period. Although the dynamics of infection within the study cohort mirrors local incidence, the figure of SARS-CoV-2 infections in children and adolescents appears to be high. Reported contact with SARS-CoV-2-infected individuals in the same household carries a high risk of infection.

## 1. Introduction

SARS-CoV-2 infections in children are diagnosed less frequently than in adults [1,2]. Reasons for this might include high percentages of oligo- or asymptomatic infections [3] but also undertesting, especially in young children who often get quarantined with their infected parents without being tested themselves.

Seroconversion studies, however, show that the infection rate in children and adolescents is probably similar to adults. Population-based approaches have shown very different percentages of seroconversion in different parts of the world and vary within countries [4,5,6]. Therefore, the role of children in spreading the infection is still not well understood. In this cohort study, prospective, longitudinal, and population-based data on seroconversion rates in asymptomatic children and their parents were gathered to improve the understanding of infection chains and help to improve infection control measures.

## 2. Materials and Methods

Asymptomatic children and adolescents (and their parents) who attended outpatient pediatric practices from June 2020 to February 2021 in the Ruhr region, located in Western Germany, for scheduled mandatory routine examinations (U—untersuchungen) from 6 months to 18 years of age were invited to participate in the study. None of the participants were vaccinated against SARS-CoV-2 at the time of the study. The participants and their parents were asked to fill in a tablet-based questionnaire available in 5 different languages. The questionnaire included questions about former SARS-CoV-2 infection and exposure to SARS-CoV-2-infected individuals. Serum samples were analyzed for SARS-CoV-2 IgM and IgG antibodies using the Roche Elecsys^®^ IgM and IgG qualitative antibody assay (directed at the nucleocapsid antigen (N—antigen)).

### 2.1. Sources of Additional Data

Case counts of acute SARS-CoV-2 infections defined by positive PCR were registered in Germany on a weekly basis by the Robert-Koch Institute (RKI) and stratified by age and county [7]. Here, time series of case counts for the counties of Gütersloh, Bochum and Herne were used to account for the three most frequently represented counties in the CorKid survey. Regarding Gütersloh, population data are available for age groups “0–5 ys”, “6–17 ys 18”, whereas for Bochum, information on three age groups “0–5 ys”, “6–9 ys” and “10–17 ys” are available. Regarding Herne, population data for age groups “0–2 ys”, “3–5 ys”, “6–9 ys”, “from 10 to less than 15”, “from 15 to less than 18” are available. We assumed uniformly distributed population sizes within each age group to derive an approximate age distribution per year of life.

### 2.2. Mathematical Methods

To directly compare the observed seroprevalence in the CorKid cohort with the confirmed cases reported by the Robert-Koch Institute (RKI), an age-standardization procedure was used. Both seroprevalence as well as confirmed cumulative cases were given monthly per year of life. Population data were retrieved for the three counties represented the most in the CorKid cohort and were used to calculate age-stratified per capita registered cumulative case counts. Then, using the age distribution of the CorKid cohort as weights, standardized total per capita counts were calculated to allow for a direct comparison with the observed total seroprevalence.

Confidence intervals (CIs) for the monthly observed total seroprevalence were computed as exact 95% CIs for proportions to obtain a monthly confidence band. Furthermore, the ratio of the total seroprevalence to the standardized cumulative cases was calculated per month using the lower and upper limits of the confidence band for the seroprevalence to obtain a confidence band for the ratio.

Alternatively, a logistic function, $LL2.2\left(t\right)=\frac{1}{1+\exp\left(b\left(\log\left(t\right)-e\right)\right)}$, was fitted to the observed seroprevalence time course to account for the fact that seroprevalence monotonously increases with time. The obtained prediction curve along with the 95% confidence band was additionally used to calculate the ratio to the registered cases.

Mathematical analysis was performed using statistical programming language R [8]. Particularly, the logistic function was fitted using the drc-package [9], which is a least-squares-based curve-fitting routine offering in-build functions to be fitted. Specifically, LL2.2. is a logistic function with a minimum 0 and maximum 1 tailored to model binomial response. Parameters *b* and *e* serve as dummy parameters without significance for the purpose of total curve evaluation. The package also offers the computation of confidence bands based on the delta method, which was used here to quantify the precision of the estimated ratio of total seroprevalence to standardized cumulative cases.

## 3. Results

### 3.1. Descriptive Analysis

**Figure 1:**
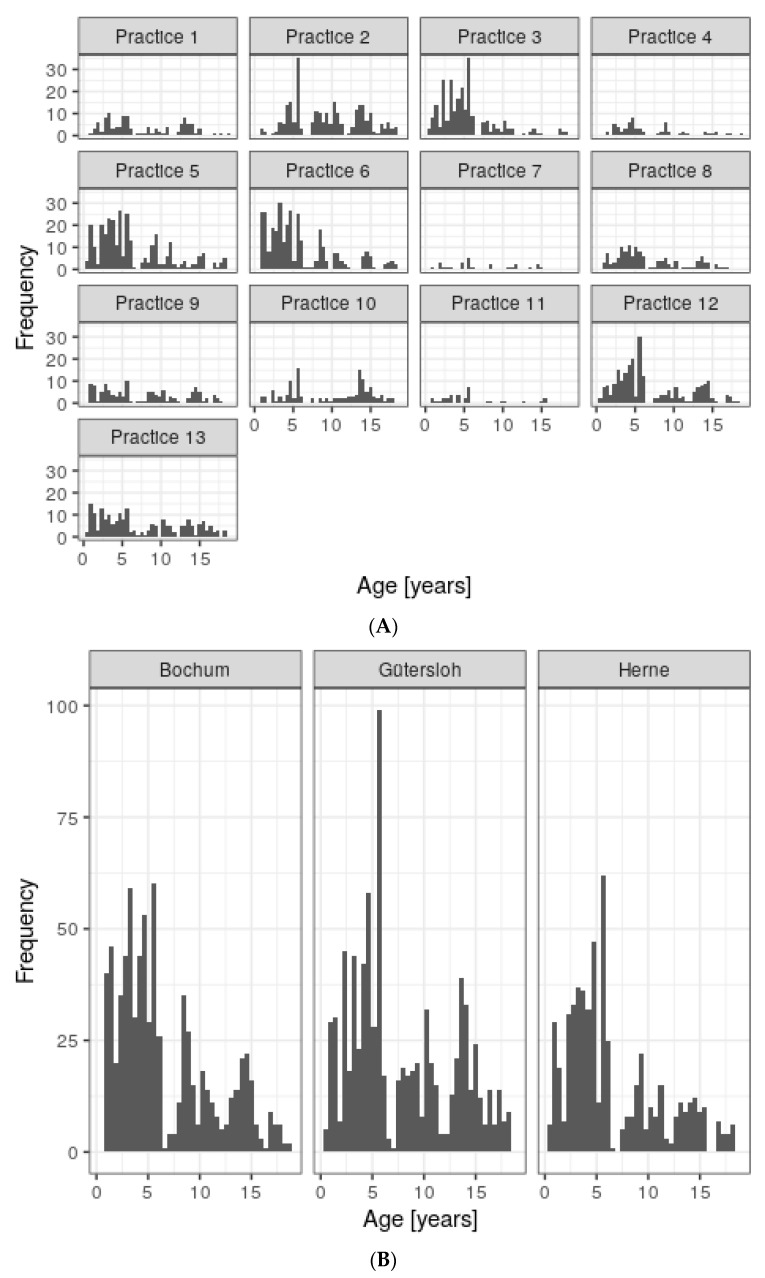
(**A**) Age distribution within the CorKid cohort. (**B**) Distribution per county.

**Figure 2:**
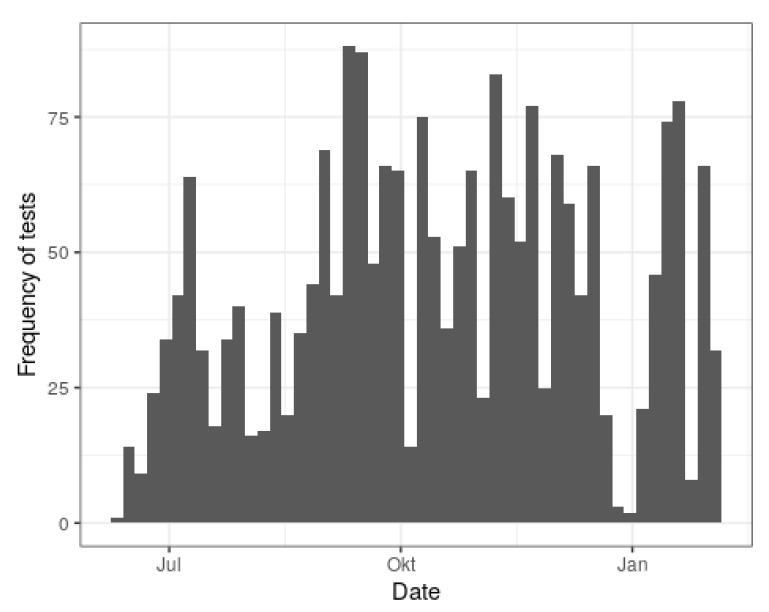
Test frequency by time over the study period from June 2020 to February 2021.

The majority of the 2184 children tested for SARS-CoV-2 antibodies within the scope of the CorKid survey come from Gütersloh, followed by Herne, Bochum and two further minor represented counties (cf. Table 1). The included areas of the minor represented counties strongly share social space with the adjacent major represented counties, which justified the choice to join them. Missing data and drop-outs due to the failure of antibody measurement because of insufficient amount of serum left n = 2045. In total, 13 pediatric practices were involved. The age distributions stratified for both practice as well as county are depicted in Figure 1. The shapes of the apparently quadro-modal distributions reflect the typical age at mandatory routine investigations and are similar across the practices and counties, only differing in magnitude. Generally, younger children below an age of 6 years old are over-represented because of the specific study design. The test frequency over time remained constant to a good approximation, except for one marked pause during the Christmas period and a marked peak during one September week (Figure 2). Of note, the survey started mid-June 2020 and ended mid-February 2021, which explains the seemingly lower frequency during these months. Of the 55 children with seroconversion, 23 (41%) recalled symptoms of infection during the preceding three months, whereas 32 did not.

### 3.2. Quantitative Analysis

**Figure 3:**
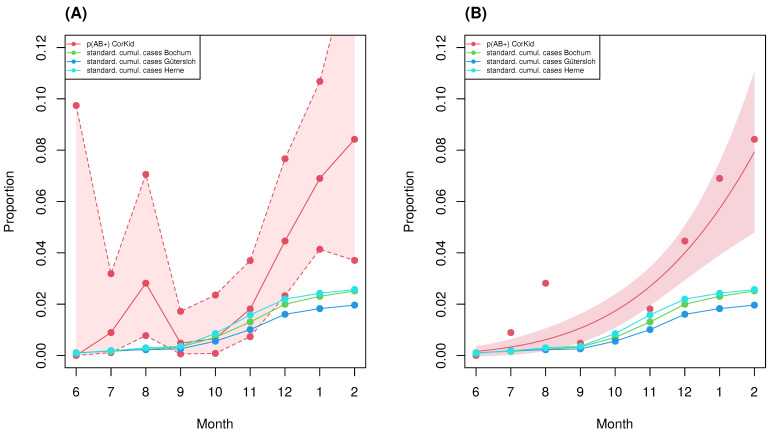
Time series of observed seroprevalence (proportion of seropositive patients) from the Corkid study and official RKI-registered age-standardized per capita cumulative cases for the three counties relevant for the study. (**A**) Plain observations with 95% confidence interval. (**B**) Model prediction from a logistic curve fitted to the observed seroprevalence.

The observed seroprevalence is described in monthly intervals from June 2020 through February 2021 (the study period), to assess the temporal behavior. The same holds for the cumulative case numbers publicly reported by the Robert-Koch Institute (RKI) for the three most represented counties. The overall proportion of seropositive children (index children) observed in a first examination in the CorKid survey amounts to 2.5% (52/20930) (note the drop-out due to failures of testing). Thereof, 36 children were examined a second time after a median term of 197 days. Thereby, an additional number of 3 children tested seropositive, such that total seropositivity amounts to 2.6% (55/2090). However, an approximately exponential increase in seroprevalence from about zero to roughly 8% within the CorKid cohort during the study period can be observed (Figure 3).

**Figure 4:**
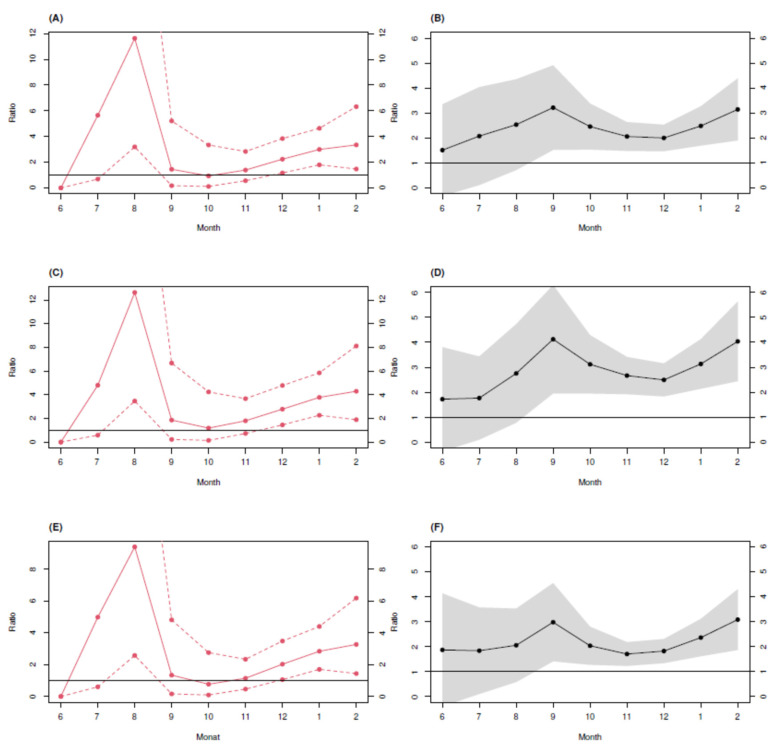
Ratio of observed seroprevalence from the Corkid study to the RKI-confirmed cumulative cases by time. RKI data [7] are age-standardized. Left column (**A**,**C**,**E**): ratio based on plain seroprevalence observations. Right column (**B**,**D**,**F**): ratio based on the fitted logistic curve. (**A**,**B**): Bochum. (**C**,**D**) Gütersloh. (**E**,**F**) Herne.

The cumulative number of cases reported by the RKI [7] were age standardized with respect to the age distribution observed for the CorKid cohort to allow for direct comparisons. It turns out that seroprevalence towards the end of the study period (February 2021) was three-fold higher for Bochum and Herne and four-fold higher for Gütersloh, compared to the corresponding standardized reported cumulative case number (Figure 4. In the beginning of the study period (June 2020), we observed a ratio of seroprevalence to registered cases of only roughly two for all three counties, suggesting that children’s contributions to the epidemic are increasingly under-represented in the officially reported counts.

The comparison of the seroprevalence obtained in the CorKid survey with the RKI-confirmed cumulative cases was either based on a direct ratio of the observed seroprevalence to the reported cumulative cases or on the ratio between the predicted seroprevalence from a nonlinear regression curve to the cumulative cases. While nonlinear regression has theoretical evidence and is, therefore, the preferred method of choice, we opted to show the results side by side (Figure 4) to allow for a complementary assessment.

## 4. Discussion

SARS-CoV-2 infections in children and adolescents have been reported with varying frequencies throughout the pandemic, ranging from 0.5 to 5% of the total infection rate [1,2,7] determined by PCR. Early seroconversion studies suggested a very low incidence of SARS-CoV-2 infection in children, followed by a rise during the second epidemic wave in schoolchildren in the UK, Switzerland and Austria [4,5,6], as well as in Germany [7].

The reported frequency of symptomatic SARS-CoV-2 infections in children and adolescents ranges from 16 to 55%, suggesting that at least half of the actual infection events pass unnoticed [4,10,11].

Asymptomatic children and adolescents were more likely to not be tested for SARS-CoV-2 before routine testing was introduced roughly from winter 2020 onward or earlier, depending on the country in Europe, and, therefore, infection chains largely remain unclear. Nevertheless, several studies describe household transmission as a major risk factor [11,12]. In line with this, in our study cohort, more than half of seropositive children had seropositive parents. Remarkably, the age of the participants did not play a crucial role in our cohort. The seroconversion rate was very similar in all age groups, as described before in a Danish population-based study [13].

The limitations of the study include a selection bias caused by different clientele in the pediatric practices which participated in the study. Furthermore, waning immunity can play a role in participants with delayed serological tests after infection, which may give rise to an underestimated seroprevalence. Finally, we assumed a sufficiently high precision of the antibody tests used. Presumably high specificity does not entail a serious limitation; however, non-optimal sensitivity, as described in other cohorts with PCR-confirmed SARS-CoV-2 infections [14], may lead to a slightly underestimated seroprevalence.

## 5. Conclusions

Seroconversion rates reveal a high percentage of undetected SARS-CoV-2 infections in children and adolescents. This substantial number of unreported cases among children should be considered in policies of preventive measures.

## Figures and Tables

**Table 1:** Summary descriptive table by groups of seropositivity (missing data are not displayed).

	0	1
	n = 2090	n = 55
Sex:		
Female	1006 (48.2%)	26 (47.3%)
Male	1083 (51.8%)	29 (52.7%)
Age group:		
[0, 6)	1193 (57.4%)	34 (61.8%)
[6, 10)	303 (14.6%)	7 (12.7%)
[10, 19)	583 (28.0%)	14 (25.5%)
County:		
Bochum	749 (35.8%)	14 (25.5%)
Gütersloh	819 (39.2%)	17 (30.9%)
Herne	522 (25.0%)	24 (43.6%)

## Data Availability

The data presented in this study are available on request from the corresponding author. The data are not publicly available due to funding restrictions.
